# 4152 Special Delivery: Home Delivery of Healthy Food to Young Women during Pregnancy

**DOI:** 10.1017/cts.2020.427

**Published:** 2020-07-29

**Authors:** Ione Locher, Marika Waselewski, Tammy Chang

**Affiliations:** 1 University of Michigan School of Medicine

## Abstract

OBJECTIVES/GOALS: Most pregnant youth (ages 14-24) gain more weight during pregnancy than recommended by clinical guidelines. We aim to describe the feasibility and acceptability of home grocery delivery of fruits, vegetables, and healthy snacks to promote healthy weight gain in this vulnerable population. METHODS/STUDY POPULATION: Participants were low-income pregnant youth in Michigan. Each participant was sent biweekly grocery deliveries consisting of $35 worth of fresh fruits, vegetables, and healthy snacks via the app-based delivery service, Shipt. Between deliveries, participants were prompted to respond to weekly text message-based surveys of a 24-hour food recall. This validated nutritional assessment quantifies consumption of fruit and vegetable servings. In addition, participants were asked to send daily photos and descriptions of foods they were eating. This study was approved by the University of Michigan Institutional Review Board. RESULTS/ANTICIPATED RESULTS: To date, 27 participants have been enrolled. Thirteen participants have completed their participation, 4.3 months on average, and were sent an average of 10 grocery deliveries each. In total, over 200 deliveries have been sent with 86% confirmed by the study participant (179/207). Additional outcomes to be assessed include: 1) text message response rates by participants and 2) content from photos and text descriptions of food eaten by participants. The 24-hour recall and text and photo messaging provided in-context data about grocery utilization. DISCUSSION/SIGNIFICANCE OF IMPACT: Grocery delivery is both feasible and acceptable to our youth participants. Use of grocery delivery constitutes a novel intervention to promote healthy weight gain in pregnancy for vulnerable populations through improving access to healthy food options.

